# Role of two RpoN in *Bradyrhizobium* sp. strain DOA9 in symbiosis and free-living growth

**DOI:** 10.3389/fmicb.2023.1131860

**Published:** 2023-02-16

**Authors:** Jenjira Wongdee, Pongdet Piromyou, Pongpan Songwattana, Teerana Greetatorn, Neung Teaumroong, Nantakorn Boonkerd, Eric Giraud, Nico Nouwen, Panlada Tittabutr

**Affiliations:** ^1^Institute of Research and Development, Suranaree University of Technology, Nakhon Ratchasima, Thailand; ^2^School of Biotechnology, Institute of Agricultural Technology, Suranaree University of Technology, Nakhon Ratchasima, Thailand; ^3^IRD, Plant Health Institute of Montpellier, UMR-PHIM, IRD/CIRAD/INRAE/Université de Montpellier/SupAgro, Montpellier, France

**Keywords:** *Bradyrhizobium*, RpoN, nitrogen fixation, free-living, symbiosis, nodulation, extracellular polysaccharide

## Abstract

RpoN is an alternative sigma factor (sigma 54) that recruits the core RNA polymerase to promoters of genes. In bacteria, RpoN has diverse physiological functions. In rhizobia, RpoN plays a key role in the transcription of nitrogen fixation (*nif*) genes. The *Bradyrhizobium* sp. DOA9 strain contains a chromosomal (c) and plasmid (p) encoded RpoN protein. We used single and double *rpoN* mutants and reporter strains to investigate the role of the two RpoN proteins under free-living and symbiotic conditions. We observed that the inactivation of *rpoNc* or *rpoNp* severely impacts the physiology of the bacteria under free-living conditions, such as the bacterial motility, carbon and nitrogen utilization profiles, exopolysaccharide (EPS) production, and biofilm formation. However, free-living nitrogen fixation appears to be under the primary control of RpoNc. Interestingly, drastic effects of *rpoNc* and *rpoNp* mutations were also observed during symbiosis with *Aeschynomene americana*. Indeed, inoculation with *rpoNp*, *rpoNc*, and double *rpoN* mutant strains resulted in decreases of 39, 64, and 82% in the number of nodules, respectively, as well as a reduction in nitrogen fixation efficiency and a loss of the bacterium’s ability to survive intracellularly. Taken together, the results show that the chromosomal and plasmid encoded RpoN proteins in the DOA9 strain both play a pleiotropic role during free-living and symbiotic states.

## 1. Introduction

Symbiotic bradyrhizobia play an important role in legume growth and development under nitrogen-limiting conditions *via* their active conversion of atmospheric N_2_ into plant-assimilable ammonium using the nitrogenase enzyme complex. The enzyme complex is structurally highly conserved throughout nitrogen-fixing bacteria (Dixon and Kahn, 2004) and is composed of 2 subunits: Fe-protein (NifH) and MoFe-protein (NifD/K). Besides the genes expressing the three structural proteins of nitrogenase (*nifH*, *D*, and *K*), many other *nif* genes are required for the formation of co-factors and/or the assemblage of an active nitrogenase. The expression of *nif* genes is managed *via* hierarchically organized regulatory genes. Their cooperative action allows the bacteria to sense the environmental conditions and to regulate the *nif* gene expression levels required for optimal nitrogen fixation (Lindström and Mousavi, 2019).

In most nitrogen fixing bacteria, the regulation of *nif* gene transcription depends on the NifA-RpoN regulatory cascade. Consequently, promoters of *nif* genes contain both RpoN and NifA binding sites. Promoters recognized by primary sigma factors are characterized by having a consensus sequence [i.e., an upstream activating sequence (UAS)], which contains an unvarying GG at-24 and GC at-12 upstream of the transcriptional start site (5′-T**GG**CAC-N_5_-TT**GC**-3′) (Salazar et al., 2010). RpoN is an alternative sigma factor that is found in a broad range of bacteria and directs gene expression *via* enhancement of the activity of RNA polymerase. In the majority of bacteria, RpoN has been shown to be involved in regulating the transcription of genes involved in nitrogen regulation (*ntr*), dicarboxylic regulation (*dct*), and nitrogen fixation (*nif* and *fix*). However, in *Escherichia coli* K-12 and *Clostridium beijerinckii*, the RpoN regulon also includes genes implicated in motility, carbon metabolism, and nitrogen assimilation (Belik et al., 2008; Hocq et al., 2019). Moreover, in the pathogenic bacteria *Burkholderia cenocepacia* and *Vibrio cholerae*, RpoN has been shown to be essential for colonization of the host by regulating biofilm formation and type VI secretion (T6SS), respectively (Dong and Mekalanos, 2012; Fazli et al., 2017).

In rhizobia, various roles of RpoN have been reported during free-living and symbiotic states. A *rpoN* mutant of the symbiotic bacterium *Sinorhizobium meliloti* forms Fix^−^ nodules on alfalfa and cannot grow on C4-dicarboxylates, which are plant-supplied substrates for nitrogen fixation in indeterminate nodules (Ronson et al., 1987). Another example, *Bradyrhizobium diazoefficiens* (previously name *B. japonicum*) strain USDA110 contains two *rpoN* genes, and inactivation of both copies results in a Fix^−^ phenotype in soybean plants, but single mutants are completely functional in symbiosis. In contrast to *S. meliloti*, the two RpoNs of *B. diazoefficiens* USDA110 are not involved in the ability to use C4-dicarboxylates as well as a large array of nitrogen sources under free-living conditions but do play a role in nitrate assimilation (Kullik et al., 1991). Experiments with an *rpoN* mutant of *Rhizobium etli* showed that rhizobial RpoN is not obligatory for symbiotic nitrogen fixation. In this bacterium, RpoN is only important under free-living conditions, where it controls the production of melanin, the activation of *nifH*, and the metabolism of C4-dicarboxylic acids and several nitrogen sources (Michiels et al., 1998). These few examples show the different facets of the regulator RpoN’s role in the physiology of rhizobia during their free and symbiotic lives.

*Bradyrhizobium* sp. strain DOA9 is able to fix atmospheric dinitrogen under both free-living conditions and in symbiosis with *Aeschynomene americana* plants (Wongdee et al., 2016). Genomic analysis revealed that DOA9 contains one chromosome (c) and one symbiotic plasmid (p), which both carry nitrogen-fixing (*nif*) genes that include the nitrogenase structural genes *nifH*, *nifD*, and *nifK* and the nitrogenase regulatory genes *nifA* and *rpoN* (Okazaki et al., 2015). Our previous study showed that *nif* genes from both chromosome and plasmid are required for the nitrogenase activity during the symbiosis with *A. americana* and that only the *nif* genes located on the chromosome (*nifDKc* and *nifAc*) are required for the nitrogenase activity in the free-living state. Interestingly, full symbiotic nitrogenase activity of DOA9 with *A. americana* requires the presence of NifDK encoded from both chromosome and plasmid, whereas inactivation of one of the *nifA* copies has no effect on the symbiotic nitrogen fixation, indicating that the two NifA proteins are functionally redundant (Wongdee et al., 2016, 2018). Besides two copies of *nifA*, the DOA9 strain also contains a chromosomal and plasmid-encoded RpoN protein. Thus, it is interesting to examine the role of these two copies (*rpoNc* and *rpoNp*) in the controlling of *nif* gene expression and the activation of other metabolic functions under symbiotic and free-living conditions.

We investigated the expression profile of *rpoNc* and *rpoNp* during nitrogen fixation under symbiotic and free-living conditions by constructing a translational *gusA* fusion with the promoter sequence of each *rpoN* gene. In addition, single and double *rpoN* mutants in DOA9 were constructed to analyze the contribution of each regulatory protein during symbiosis with *A. americana* and their roles in carbon and nitrogen utilization, extracellular polysaccharide (EPS) production, and biofilm formation under the free-living growth. Finally, the expression level of several *nif* genes in different mutant backgrounds (Δ*rpoNc*, Δ*rpoNp*, and Δ*rpoNp*::Ω*rpoNc*) was analyzed to identify whether these genes are controlled by RpoNc or RpoNp under symbiotic and free-living conditions. The experiments revealed that in *Bradyrhizobium* sp. strain DOA9, RpoN plays a critical role in the regulation of *nif* gene expression and the activation of several other metabolic functions, which significantly contributes to the nodulation and nitrogen fixation efficiency of DOA9 under both symbiosis with *A. americana* and free-living growth.

## 2. Materials and methods

### 2.1. Bacterial strains and culture conditions

*Bradyrhizobium* sp. DOA9 and its derivative strains were obtained from Applied Soil Microorganism Laboratory, School of Biotechnology, Suranaree University of Technology, Thailand. These bacterial strains were routinely grown in liquid yeast extract mannitol (YEM) (Vincent, 1970), arabinose-gluconate (AG) (Sadowsky et al., 1987), and buffered nodulation (BNM-B, minimal medium) (Renier et al., 2011) at 30°C for 5 days and maintained on YEM agar plates. *Escherichia coli* strains were grown in Luria-Bertani (LB) medium at 37°C. When necessary, antibiotics were supplied at the following concentrations: kanamycin: 300 μg/ml; streptomycin: 300 μg/ml; nalidixic acid: 20 μg/ml; cefotaxime: 20 μg/ml.

### 2.2. Construction the reporter and *rpoN* mutant strains

To construct the promoter GusA-fusion of *rpoNc* and *rpoNp* operons, a ±400-base-pair (bp) upstream region of each *rpoN* gene was amplified by PCR (see in the primer list, Supplementary Table S1), and the corresponding DNA fragment was cloned into plasmid pVO155-*npt2*-*cefo*-*npt2*-*gfp*. This vector is a derivative of the pVO155 plasmid (Oke and Long, 1999) that cannot replicate in *Bradyrhizobium* strains and besides a promoterless *gusA* gene, contains the *gfp* gene under control of the constitutive *nptII* promoter and kanamycin and cefotaxime resistance genes (Okazaki et al., 2016). The plasmid was introduced into *Bradyrhizobium* sp. DOA9 *via* conjugation and clones in which the plasmid was inserted into the target region *via* single crossing over were isolated by selection on YM plates containing nalidixic acid and cefotaxime. Isolated clones were verified by sequencing, and good clones were named *PmrpoNc* and *PmrpoNp* (a schematic overview of the *pVO155*-*npt2*-*cefo*-*npt2*-*gfp* construct integrated into the target region of the GusA reporter strains is given in Supplementary Figure S1).

To construct the insertional mutants of *rpoNc* and *rpoNp* (DOA9Ω*rpoN* mutants), a ±300-bp internal sequence fragment of each *rpoN* gene was amplified by PCR and cloned into plasmid pVO155-*npt2*-*cefo*-*npt2*-*gfp*.

To obtain a complete gene deletion of *rpoNc* and *rpoNp* (DOA9∆*rpoN* mutants), overlapping extension PCR was used to fuse fragments containing upstream and downstream regions (around 700 bp) of each *rpoN* gene (see in the primer list, Supplementary Table S1). Subsequently, the obtained DNA fragment was cloned into plasmid pK18*mob*-*cefo*-*sacB*. The six constructed plasmids were transferred into DOA9 by biparental mating, followed by the selection of insertional and deletion mutants as described previously (Wongdee et al., 2016, 2018). The DOA9∆*rpoNp*::Ω*rpoNc* mutant was constructed by electroporation of pVO155-*npt2-cefo-npt2-gfp* carrying an internal *rpoNc* region into competent DOA9∆*rpoN*p cells. The electrocompetent cells of DOA9∆*rpoNp* were prepared by culturing the cells in liquid YEM medium at 30°C, 200 rpm until the bacterial cells reached 0.4–0.6 of OD_600_. Cells were harvested by centrifugation at 4,000 rpm for 15 min and washed twice with cold water and finally resuspended with 10% of cold glycerol and stored at –80°C before begin used. The electroporation was carried out with capacitor settings of 25 μF, 100 Ω, and a voltage of 1.75 kv/cm for 0.2-cm cuvettes. Subsequently, the mutant bacteria were selected on a YEM agar plate with appropriate antibiotics.

### 2.3. Colony morphology and growth phenotypic analysis under free-living conditions

The bacterial colony morphology of *rpoN* mutants (DOA9∆*rpoNc*, DOA9∆*rpoNp*, and DOA9∆*rpoNp*::Ω*rpoNc*) was compared to that of wild-type DOA9 (DOA9WT) when grown on different culture media. Cells of each strain were streaked on the quadrants of culture medium plates of YEM, AG, and BNM-B (supplemented with 20 mM succinate) with and without appropriate concentrations of antibiotics, and the plates were incubated for 7 days at 30°C. After incubation, the single colonies on medium plates were observed under light microscopy and measured for colony size.

0.3% agar (w/v) in AG medium was used for motility tests. The DOA9WT and derivative strains were grown as starter cultures for 5 days in YEM broth at 30°C. Cultures were washed twice with BNM-B medium without carbon and nitrogen source and the cell pellet was solubilized in BNM-B medium to an optical density of 1 at 600 nm (OD_600_). A sterilized toothpick was dipped into the bacterial solution and subsequently stabbed into the AG medium with 0.3% agar (w/v). After 7 days of incubation at 30°C, the bacterial motility was determined by measuring the diameter of the colony.

To analyze the ability to utilize different carbon (C) and nitrogen (N) sources, the bacterial starter culture of each strain was prepared as described above. To examine the utilization of C (20 mM) and N (10 mM) sources, 1 ml of culture medium to be tested (single C or N) was inoculated with 5% (v/v) starter culture. The medium was supplemented with ammonium nitrate (NH_4_Cl) for carbon utilization tests and with glucose for the nitrogen utilization test. After 10 days at 30°C (150 rpm), bacterial growth was determined by measuring the culture turbidity at 600 nm (Lundgren et al., 2015).

### 2.4. Extracellular polysaccharide production

To investigate the total EPS production by DOA9 and derivatives, 1% (w/w) of starter culture was inoculated in fresh YEM broth medium. After 5 days at 30°C (150 rpm), bacterial cultures were centrifuged for 15 min at 8,000× *g* in a table centrifuge (Thermo Scientific™, Sorvall™ Legend™ XRF Centrifuge). The culture supernatant was collected, and ice-cold ethanol [95% (v/v) was added until a final concentration of 35% (v/v)]. After vigorously mixing the mixture, it was incubated overnight at 4°C. Precipitated EPS was collected by centrifugation at 8000 × *g* for 20 min at 4°C. Residual ethanol was removed by drying the pellet for 5 h in a laminar flow hood and subsequently the EPS was dried by lyophilization. The weight of the lyophilized EPS pellet was measured and the amount of EPS in the culture supernatant was expressed as the mg dry weight per culture volume [this protocol was modified from Bomfeti et al., 2011 and Castellane et al., 2015].

### 2.5. Determination of biofilm formation

Biofilm formation was measured by the ability of bacterial cells to adhere to the surface of polypropylene culture tubes (50 ml; Greiner bio one International GmbH, Kremsmünster, Austria) using a modification of previously reported protocols (Castellane et al., 2015; Ghosh and Maiti, 2016). Five-day-old cultures of DOA9WT, DOA9∆*rpoNc*, DOA9∆*rpoNp*, and DOA9∆*rpoNp*::Ω*rpoNc* grown in liquid YEM medium with appropriate antibiotics were harvested and washed as described above, and cells were finally dissolved in BNM-B medium until an OD_600_ of 1 was obtained. Next, 1.5 ml of each cell solution was used to inoculate 13.5 ml of BNM-B medium containing 20 mM succinate as the sole C source. After inoculation, the tubes were incubated for 14 days at 28°C without shaking.

To circumvent precipitation of cells, cultures were briefly mixed every 2 days. Bacterial cultures were discarded, and the tubes were washed three times with distilled water. After removing all the liquid residues, a 0.1% (w/v) aqueous solution of crystal violet was added and incubated for 15 min at room temperature. The dye was carefully removed, and tubes were rinsed three times with distilled water. The empty tubes were air dried for 1 h before adding 15 ml of absolute ethanol. To determine the biofilm formation of each bacterial strain, the absorbance of the ethanol solution was measured at 570 nm and calculated as presented by Soto et al. (2006).

### 2.6. Free-living nitrogen fixation of WT DOA9 and *rpoN* mutants

Bacterial cells were harvested from YEM broth after 5 days of cultivation and washed twice with liquid BNM-B medium without C and N sources. The washed cells were inoculated into 150-ml glass bottles containing 50 ml of BNM-B medium (Wongdee et al., 2018). After closure of the bottle with an airtight stopper, 15 ml of acetylene (10% final concentration) was injected, and the cultures were incubated at 30°C without shaking. Every 2 days after inoculation, a 1-ml air sample was taken to determine the nitrogenase enzyme activity as described previously (Renier et al., 2011). The nitrogen fixation activity was calculated from three independent cell cultures. To determine the number of viable cells in the culture at the time of the determination of the nitrogenase enzyme activity, a sample from the culture was taken to determine the colony forming units (CFU on YEM agar plates).

### 2.7. Symbiotic interaction of DOA9 and *rpoN* mutant strains with *Aeschynomene americana*

The symbiotic interaction of wild-type *Bradyrhizobium* DOA9 and derivatives with *A. americana* was analyzed as described by Wongdee et al. (2016, 2018). At 20 days post-inoculation (dpi), the numbers of nodules on the roots were determined, and the nitrogenase enzyme activity of the plants was measured using the acetylene reduction assay (ARA). For cytological analysis, fresh nodules were sliced into 30-micron sections using a vibratome (Leica VT1000S Vibrating blade microtome, United States). Nodule sections of plants inoculated with DOA9-*PmrpoN* strains were stained with X-gluc (Bonaldi et al., 2010). After staining, the sections were mounted on microscope slides and observed by light microscopy. Nodule section of plants inoculated with *rpoN* mutants were stained with Live-Dead staining reagent (Invitrogen), after which they were analyzed using confocal microscopy as described by Okazaki et al. (2016).

### 2.8. RNA extraction, cDNA synthesis, and qRT-PCR analysis

qRT-PCR experiments were carried out to determine the expression of nitrogen-fixing genes in *rpoN* mutants under free-living and symbiotic conditions. To this end, RNA was extracted from cell cultures grown under *nif*-gene-inducing conditions or from nodule bacteroides. After DNAse treatment of the extracted RNA and purification using mini-prep kits (Qiagen, Valencia, CA, United States), the iScript™ Reverse transcription Supermix for RT-qPCR (Bio-Rad Hercules, CA, United States) was used to synthesize cDNA. For the analysis of gene expression, a 50-ng cDNA sample was mixed with specific primers (Wongdee et al., 2018) in PowerUP^Tm^ SYBRTM Green master mix buffer (Applied Biosystems, US & Canada) and analyzed by qPCR using an annealing temperature of 53°C. The relative expression of target genes in the different samples was determined with QuantStudio Design & Analysis Software from Applied Biosystems using the expression of the housekeeping gene, 16sRNA, as a standard.

### 2.9. Identification of putative RpoN interacting proteins

Proteins interacting with sigma54 transcription factors have been shown to contain an RNA polymerase sigma factor 54 interaction domain that in PROSITE has been defined as an IPR002078 domain. Using this number and the search option in the MAGE website^1^, we have analyzed the chromosome and plasmid of the *Bradyrhizobium* sp. DOA9 strain for the presence of proteins containing an IPR002078 domain.

## 3. Results

### 3.1. Genomic organization and phylogenetic analysis of the two *rpoN* genes in *Bradyrhizobium* sp. DOA9

**Figure 1:**
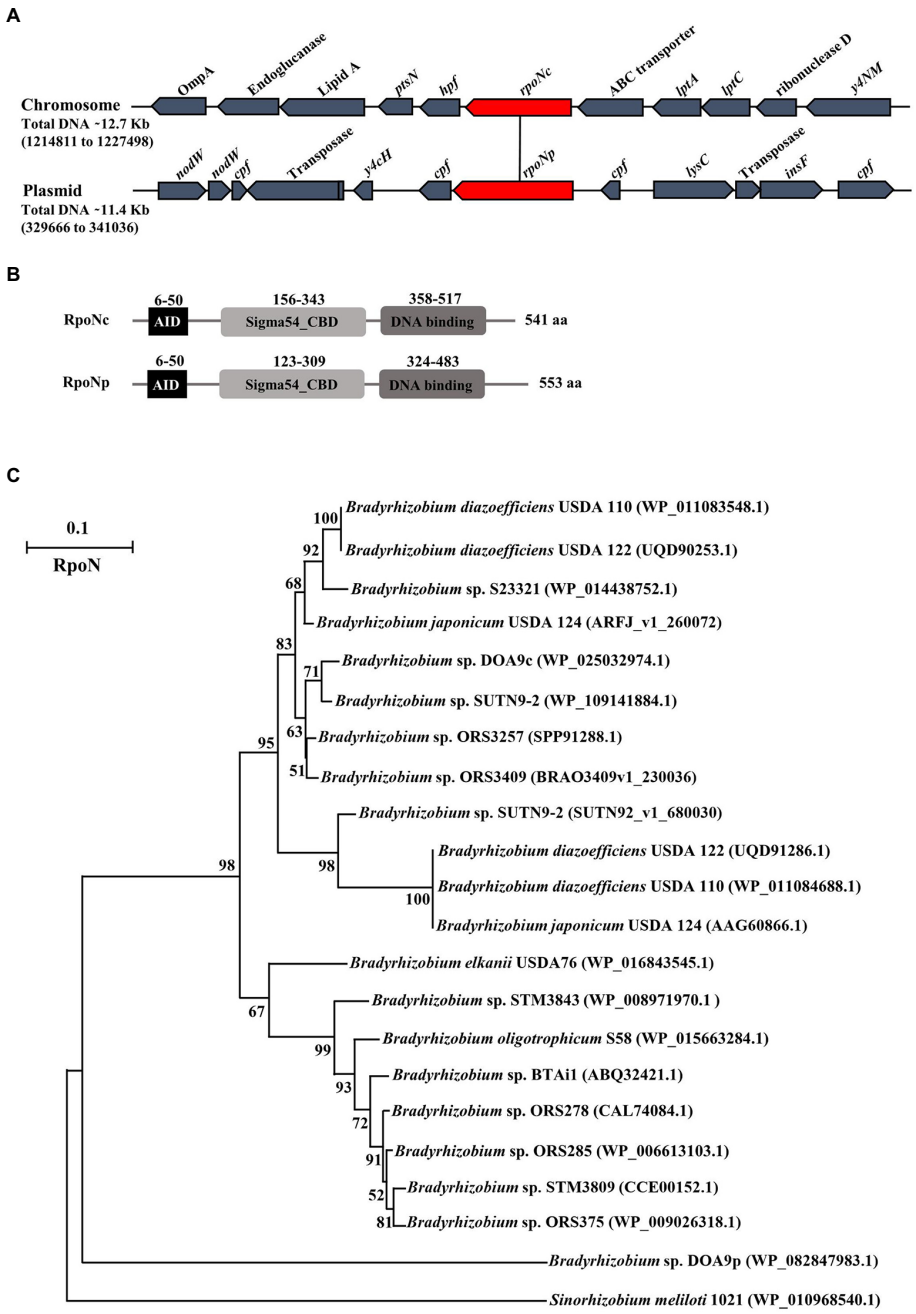
Diagram of the chromosomal and plasmid region containing *rpoN* in *Bradyrhizobium* sp. strain DOA9 **(A)**. Different protein domains present in chromosomal and plasmid encoded RpoN; AID (activator initiation domain), CBD (core binding domain), and DNA binding domain **(B)**. Phylogenetic analysis of RpoN from various *Bradyrhizobium* strains using the maximum-likelihood method with 1,000 bootstrap replicates. *Sinorhizobium meliloti* 1021 RpoN was used as outgroup protein **(C)**.

Genomic analysis showed that *Bradyrhizobium* sp. DOA9 contains two regulatory *rpoN* genes. One of the genes is localized on the chromosome (*rpoNc*), whereas the other copy is localized on the symbiotic plasmid (*rpoNp*) (Figure 1A). The *rpoNc* gene is located downstream of the *lptCA* genes that encode for an ABC transporter system involved in the transportation of lipopolysaccharides across the inner membrane. It is also upstream of the genes *hpf* and *ptsN*. The *hpf* gene encodes for a ribosome hibernation promotion factor that promotes the formation of inactive 100S ribosomes complexes during the stationary phase, while the *ptsN* gene encodes for a phosphoenolpyruvate-dependent phosphotransferase, which is involved in carbon and nitrogen metabolism (Jin et al., 1994; Potvin et al., 2008).

The inactivation of the *hpf* gene (insertional mutant) showed that this gene may function independently from *rpoN* as it does not have an impact on the nitrogenase enzyme activity under symbiotic conditions, in contrast to inactivation of *rpoN* (Supplementary Figure S2). On the plasmid, the *rpoNp* gene is located between small genes encoding for conserved proteins of unknown function (*cpf*). Interestingly, transposase genes that are normally responsible for gene transposition in bacterial genomes (Saier et al., 2017) were found up and downstream of *rpoNp*.

RpoN encodes for a sigma 54 factor that enhances the activity of RNA polymerase during transcription initiation of specific genes. The two putative RpoN proteins in DOA9 slightly differ in length [541 amino acids (aa) for RpoNc versus 553 aa for RpoNp] and display 55.82% aa identity (Figure 1B). Both contain the following: (i) an N-terminal activator interacting domain (AID), which is necessary for activator interaction (Supplementary Table S2); (ii) a C-terminal DNA binding domain (DBD), which is composed of a putative helix-turn-helix motif including the highly conserved region known as the RpoN box (−12, −24 promoter binding site); and (iii) the core enzyme binding domain (CBD) containing a classical sigma 54 motif that is predicted to form a helix-turn-helix motif that may represent another DBD. Analyzing bradyrhizobial genomes present in the magnifying genomes database, a platform of microbial genome annotation and analysis (see footnote 1) showed that the majority of *Bradyrhizobium* strains contain one copy of RpoN and only some (*Bradyrhizobium* USDA 110, USDA 124, USDA 122, and SUTN9-2) contain two *rpoN* genes that both are located on their chromosome. Phyhylogenetic analysis showed that all the RpoN sequences of non-photosynthetic bradyrhizobia are located in the same clade and separated from photosynthetic bradyrhizobia, whereas the DOA9-RpoNp sequence represents an outgroup protein (Figure 1C). Closer examination of the RpoNc and RpoNp sequences showed that the amino acid sequences of the AID, DBD, and CBD domains are relatively conserved, and that the largest differences are observed in the region between AID and CBD (RpoNp contains two large amino acid deletions) and that the RpoNp protein contains an C-terminal extension (Supplementary Figure S3). These relatively large differences between RpoNp and RpoNc of DOA9 suggest that they might have different regulatory functions.

### 3.2. Expression of *rpoNc* and *rpoNp* under free-living and symbiotic conditions

**Figure 2:**
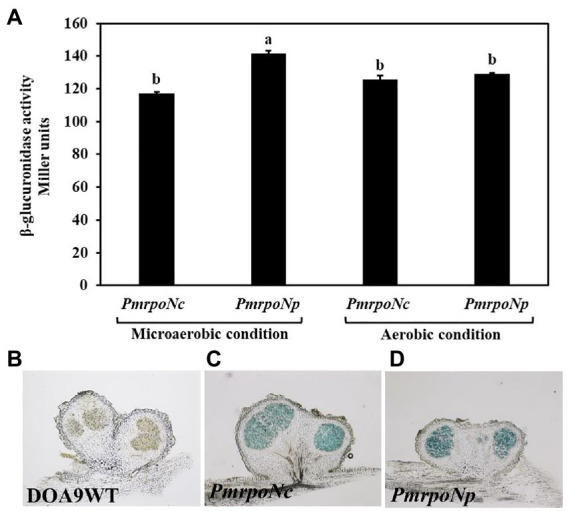
Expression of chromosomal and plasmid encoded *rpoN* under free-living and symbiotic conditions. *PmrpoNc* and *PmrpoNp* GusA reporter strains were grown under microaerobic and aerobic conditions and after 7 days of incubation, and *β*-glucuronidase activity (in Miller units) was determined. Data sets represent mean values of five replications, and statistically significant differences (ANOVA Tukey test; *p* < 0.01) are indicated **(A)**. *β*-glucuronidase activity in nodules of *Aeschynomene americana* plants inoculated with the WT and *PmrpoNc* and *PmrpoNp* GusA reporter strains at 14 dpi **(B–D)**. *β*-glucuronidase activity in nodule slices was determined by staining with X-gluc for 15 min.

To measure the expression of chromosomal and plasmid encoded RpoN, we constructed the DOA9 GusA reporter strains *PmrpoNc* and *PmrpoNp*, respectively. Measurement of the *β*-glucuronidase activity of the two reporter strains showed that both *rpoN* genes are expressed under free-living conditions (Figure 2A). There was no statistically significant difference in *β*-glucuronidase activity between the two strains when grown under aerobic conditions [126 Miller units (*PmrpoNc*) versus 129 Miller units (*PmrpoNp*)]. However, under microaerobic conditions, the *β*-glucuronidase activity of the *PmrpoNp* strain (141 Miller units) was slightly higher than that of the *PmrpoNc* strain (117 Miller units).

To analyze expression of the two *rpoN* genes during symbiosis, the *PmrpoNc* and *PmrpoNp* GusA-reporter strains were used to inoculate *A. americana* plants. Observation of inoculated plants at 14 dpi showed that both reporter strains were able to nodulate and fix nitrogen similar to the wild-type strain (Supplementary Figure S4). This indicates that the integration of the pVO155 plasmid to create the reporter strains has no effect on the symbiotic interaction of DOA9 with *A. americana* plants. Detection of *ß*-glucuronidase activity by staining nodule sections with X-gluc showed that *rpoNc* and *rpoNp* are expressed in nodules at a similar level (Figures 2C,D).

### 3.3. Deletion of *rpoN* affects the colony morphology and growth phenotype of DOA9

**Figure 3:**
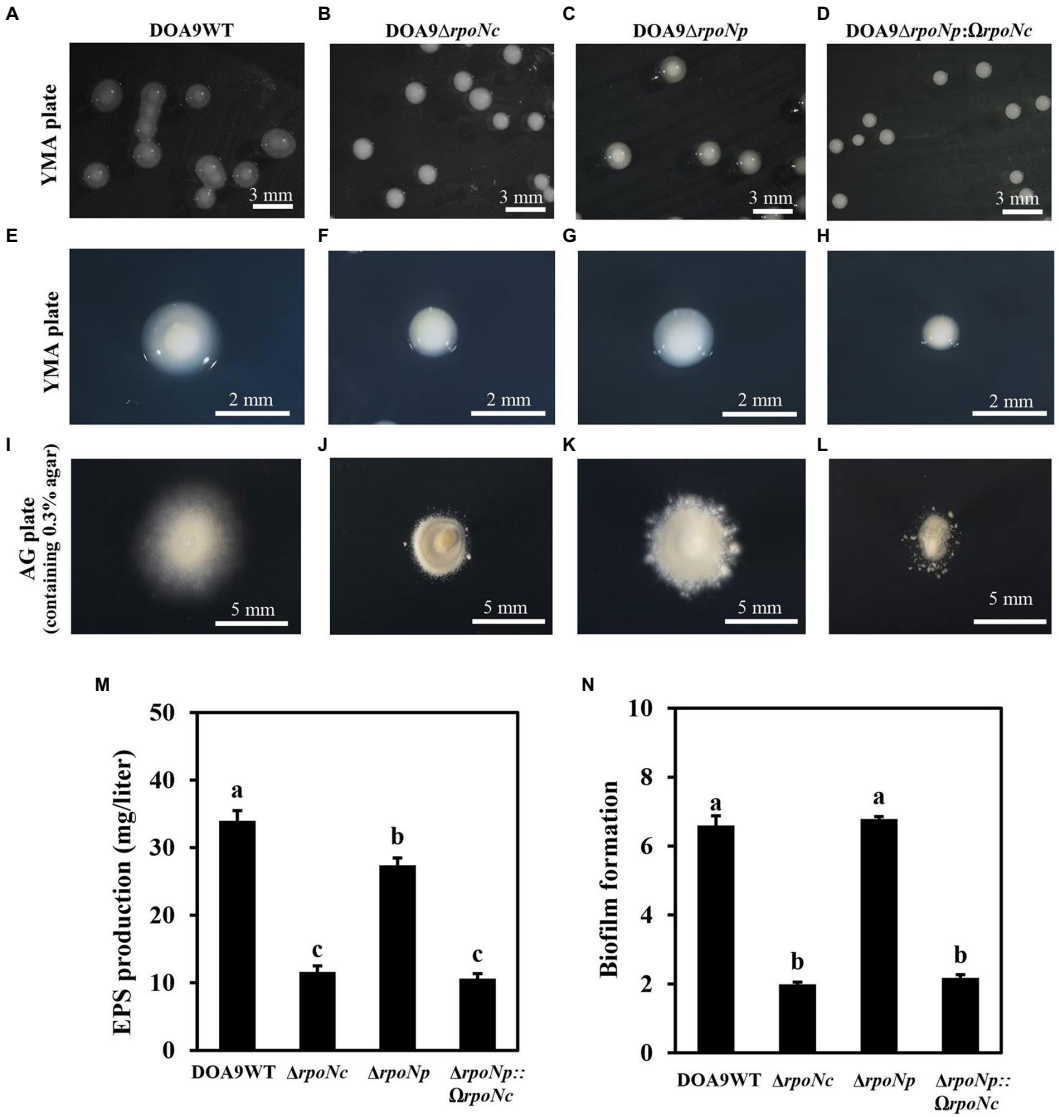
Colony morphology and growth phenotype of *rpoN* mutants of *Bradyrhizobium* sp. strain DOA9. Colony morphology of WT DOA9 **(A)** and *rpoN* mutant **(B–D)** strains after 7 days of cultivation on YEM agar plates at 28°C. The average size of single colony of WT DOA9 **(E)** and *rpoN* mutant **(F–H)** strains were observed and photographed under microscopy. Swimming motility of WT DOA9 and *rpoN* mutant strains on 0.3% BNM-B agar plates. After 7 days of incubation at 30°C the diameter of colony was measured **(I–L)**. Amount of EPS found in the bacterial culture supernatant of WT DOA9 and *rpoN* mutant cells after 7 days of incubation in YEM medium (**M**). Biofilm formation of WT DOA9 and *rpoN* mutant strains after 14 days of cultivation in BNM-B medium. The amount of biofilm formation was detected by coloring bacterial cells adhered to the plastic surface of Falcon tubes with 0.1% crystal violet and subsequently quantified by measuring the OD_570_ of the ethanol eluted crystal violet **(N)**. Data sets represent mean values of five replicates, and statistically significant differences (ANOVA Tukey test; *p* < 0.01) are indicated.

To analyze the role of the two *rpoN* genes in DOA9 under free-living and symbiotic conditions, single and double *rpoN* deletion mutants were constructed. Microscopic analysis of mutant bacteria grown on YEM agar plates showed that all *rpoN* mutants (DOA9∆*rpoNp*, DOA9∆*rpoNc*, and DOA9∆*rpoNp*::Ω*rpoNc*) produced much smaller colonies compared to the wild-type strain (Figures 3A–H). A reduced colony size was also observed when *rpoN* mutant bacteria were grown on AG and BNM-B agar medium (Supplementary Figures S5A–H). However, on the last two media, the colony size of the DOA9∆*rpoNc* and DOA9∆*rpoNp*::Ω*rpoNc* mutants was much more impacted compared to DOA9∆*rpoNp* (Supplementary Figures S5A–H). Interestingly, a similar difference in the size of the colony diameter was observed when bacteria were stabbed on AG agar medium containing 0.3% (w/v) agar, an assay routinely used to test the bacterial swimming motility (Figures 3I–L; Supplementary Figures S7A–C). These results show that inactivation of one or both *rpoN* genes has an effect on the colony morphology and swimming motility of DOA9 cells.

For other bacteria, it has been shown that inactivation mostly has an impact on the ability to use different C and N sources (Salazar et al., 2010; Lundgren et al., 2015; Hocq et al., 2019). Therefore, we analyzed the growth profile of *rpoNc* and *rpoNp* mutants on different C and N sources (Supplementary Figures S8A,B). The results show that growth of *rpoN* mutants on many different C sources is reduced compared to the growth of WT DOA9 (Supplementary Figure S8A). A similar tendency of reduced growth of *rpoN* mutants was found when amino acids were used as the N source, although the growth of the *rpoNc* and ∆*rpoNp*::Ω*rpoNc* mutants was often more impacted compared to the ∆*rpoNp* mutant (Supplementary Figure S8B). The most drastic effect on growth for all *rpoN* mutants in this study was observed when alanine, potassium nitrate, and urea were used as the N source (Supplementary Figure S8B).

### 3.4. Exopolysaccharide production and biofilm formation in DOA9 required the presence of *rpoNc*

When grown on YEM agar plates, colonies of WT DOA9 bacteria are surrounded by a “shiny” mucous layer (Figures 3A,E). This layer likely corresponds to exopolysaccharides (EPSs) produced by the bacterium. To investigate this, we determined the quantity of polysaccharides in the culture supernatant of WT and *rpoN* mutants grown in YEM medium. The DOA9WT had the highest amount of polysaccharides in the culture medium (34.6 mg/liter), and the amounts were significantly decreased to 13.5, 28, and 12 mg/liter for the DOA9∆*rpoNc*, DOA9∆*rpoNp*, and DOA9∆*rpoNp*::Ω*rpoNc* mutant bacteria, respectively (Figure 3M).

Interestingly, when we resuspended one inoculation loop of bacteria grown on YEM plates in water and performed a 15-s spin in a microcentrifuge, a large pellet was obtained with DOA9∆*rpoNc* and DOA9∆*rpoNp*::Ω*rpoNc* cells, whereas little and no pellets were obtained with the DOA9WT and DOA9∆*rpoNp* mutant cells, respectively (Supplementary Figure S5I). This indicates that besides a reduction in EPS production, inactivation of the different *rpoN* genes also has an impact on other physical characteristics of DOA9 cells. For this reason, we investigated whether *rpoN* inactivation has an impact on the adhesion of DOA9 cells to plastic surfaces (biofilm formation). Similar to the EPS production, the adherence of the *rpoNc* and ∆*rpoNp*::Ω*rpoNc* mutants to the surface of Falcon tubes and microtiter wells was clearly reduced compared to that of the WT and ∆*rpoNp* mutant (Figure 3N; Supplementary Figure S9).

### 3.5. Chromosomal encoded RpoN plays an important role in free-living nitrogen fixation

**Figure 4:**
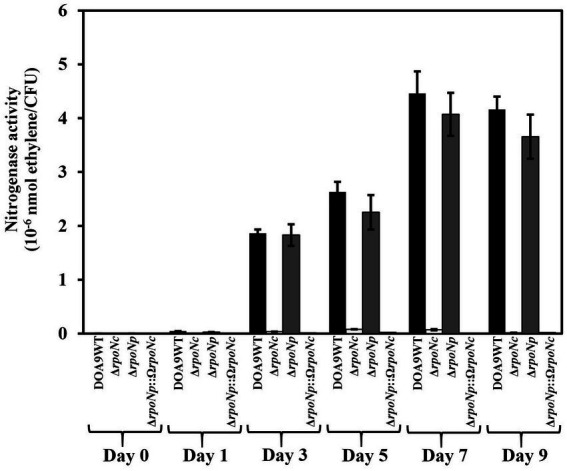
Nitrogenase enzyme activity of *Bradyrhizobium* sp. DOA9WT, DOA9Δ*rpoNc*, DOA9Δ*rpoNp* and DOA9Δ*rpoNp*::Ω*rpoNc* strains under free-living conditions. Bacterial strains were grown in BNM-B medium without N source under microaerobic conditions and at different days after inoculation the nitrogenase enzyme activity was determined using the acetylene reduction assay (ARA). For each time-point the number of living cells in the culture was determined and the nitrogenase enzyme activity was expressed as nmol ethylene/colony forming units (CFU) of the culture. Data sets represent mean values of three replications, and statistically significant differences [ANOVA Tukey test (*p* < 0.01)] are indicated.

To investigate whether RpoNc and RpoNp play a role in regulation nitrogen fixation under free-living conditions, WT and *rpoN* mutants were grown under nitrogenase-inducing conditions microaerobiosis and no assimilable nitrogen source (Wongdee et al., 2016). The nitrogenase enzyme activity was measured using ARA (Figure 4). For WT DOA9, from 3 dpi, ethylene formation could be detected. It increased until 7 dpi and subsequently remained stable. A similar ethylene formation profile was found for cultures of the *rpoNp* mutant, whereas for cultures of the *rpoN*c and double *rpoN* mutants, no ethylene formation was detected. This indicates that RpoNc plays an important role in regulating nitrogen fixation under free-living conditions.

### 3.6. Chromosomal and plasmid encoded RpoN are important for the symbiotic interaction of DOA9 with *Aeschynomene americana*

**Figure 5:**
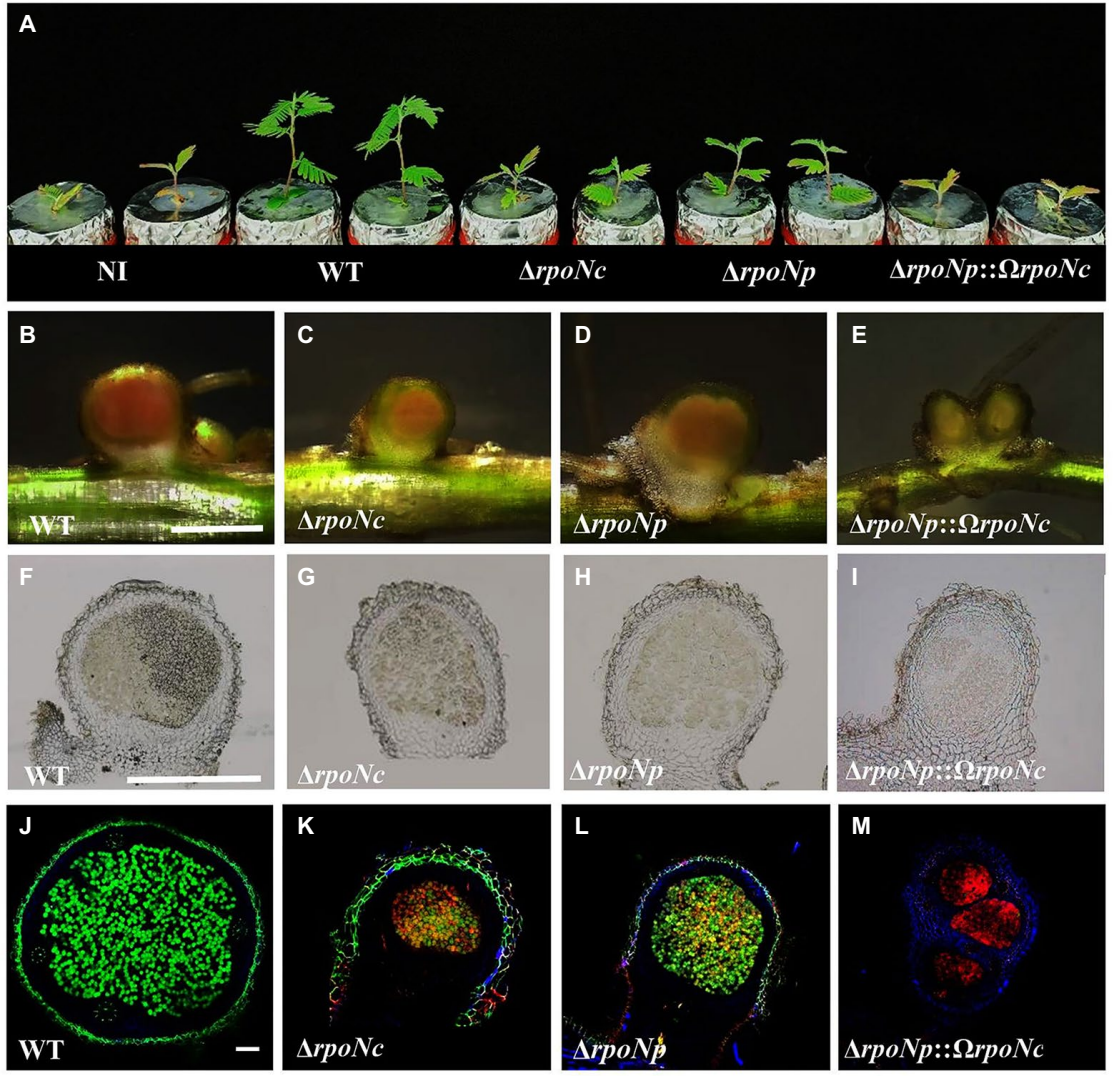
*Aeschynomene americana* (cv. Madagusga) plants grown in nitrogen-limiting conditions and inoculated with DOA9 WT and *rpoN* mutant strains at 20 dpi **(A)**, nodules as found on *A. americana* plants inoculated with WT and *rpoN* mutant strains at 20 dpi **(B–E)**. Thin nodule slices (50 μm) observed under light **(F–I)** and confocal microscopy **(J–M)**. Indicated scale bars represent 1 mm **(B–E)**, 1.5 mm **(F–I)**, and 100 μm **(J–M)**.

To analyze the role of both RpoNs in symbiosis, *A. americana* plants grown under N-limiting conditions were inoculated with the different DOA9 *rpoN* mutant strains, and at 20 dpi, the result of the interaction was characterized. Inoculation with DOA9WT stimulated the growth of *A. americana*, but plants inoculated with the *rpoN* mutants had a similar size to the non-inoculated control plants (Figure 5A). Although the sizes of plants inoculated with the Δ*rpoNp* and Δ*rpoNc* mutants were similar to that of the non-inoculated control, the leaves of the plant were less chlorotic, indicating some positive effect of inoculation. Indeed, determination of the fresh plant weight indicated a (small) stimulating effect on plant growth by the single Δ*rpoNp* and Δ*rpoNc* mutant (Table 1).

**Table 1:** Symbiotic properties of *Bradyrhizobium* sp. DOA9 wildtype and *rpoN* mutant strains.

Strains	Nitrogenase activity (nmol ethylene plant^−1^)	Plant fresh weight (mg plant^−1^)	Numbers of root nodule plant^−1^
NI	0 ± 0^e^	59.77 ± 1.35^d^	0 ± 0^e^
DOA9WT	183.43 ± 14.44^a^	200.6 ± 14.46^a^	33.44 ± 4.93^a^
Δ*rpoNc*	42.69 ± 9.51^c^	80.37 ± 4.21^c^	12.13 ± 2.85^c^
Δ*rpoNp*	105.66 ± 7.95^b^	114.60 ± 9.36^b^	22.42 ± 5.21^b^
Δ*rpoNp*::Ω*rpoNc*	4.17 ± 1.02^d^	58.83 ± 5.41^d^	6.00 ± 3.17^d^

Data are presented as mean ± standard deviation (SD; *n* = 5 plants/strain). Lower case letters indicate statistically significant differences (ANOVA Tukey test; *p* < 0.01) between analyzed strains in each parameter.

Analysis of the roots showed that all *rpoN* mutants were able to induce nodules on *A. americana* plants (Figures 5B–I), but their number was reduced in comparison to plants inoculated with DOA9WT (Table 1). In addition, the nodules on plants inoculated with DOA9Δ*rpoNp*::Ω*rpoNc* had a white appearance (Figure 5E), whereas those on plants inoculated with DOA9Δ*rpoNc* and DOA9Δ*rpoNp* had a less intense red/pink color compared to WT nodules (Figures 5B–D), suggesting that they have little or no nitrogen fixation activity. Accordingly, measuring the nitrogenase enzyme activity of the plants using the ARA showed that plants inoculated with the *rpoN* mutants had nitrogenase enzyme activity that ranged from reduced (DOA9Δ*rpoNc* and DOA9Δ*rpoNp*) to severely reduced (DOA9Δ*rpoNp*::Ω*rpoNc*) (Table 1). Taken together, the results show that both chromosomal and plasmid-encoded *rpoN* are important for symbiosis of DOA9 with *A. americana* and that there is a clear correlation between the observed stimulation of plant growth, the measured nodule number, and nitrogenase enzyme activity.

To analyze the physiological status of the bacteria present in nodules induced by the different *rpoN* mutants, 50 μm nodule sections were stained with the LIVE/DEAD™ reagent and observed by confocal microscopy. Nodules of plants inoculated with DOA9WT contained densely infected central tissue of the nodule that was colored green, indicating that the bacteria were alive (Figure 5J). Interestingly, the nodule of plants inoculated with the single *rpoN* mutants seemed to be less infected in comparison to WT nodules and contained zones with green and yellow/orange coloring bacteria. This indicated that they contained both living and dead bacterial cells (Figures 5K,L). More drastic effects were observed on the nodules formed by the double *rpoN* mutant, for which the central infected tissue was colored completely red, indicating that all bacteria were dead (Figure 5M).

### 3.7. The qRT-PCR revealed that mutation of *rpoN* genes in DOA9 strain affected *nif* genes expression under both free-living and symbiosis with *Aeschynomene americana*

**Figure 6:**
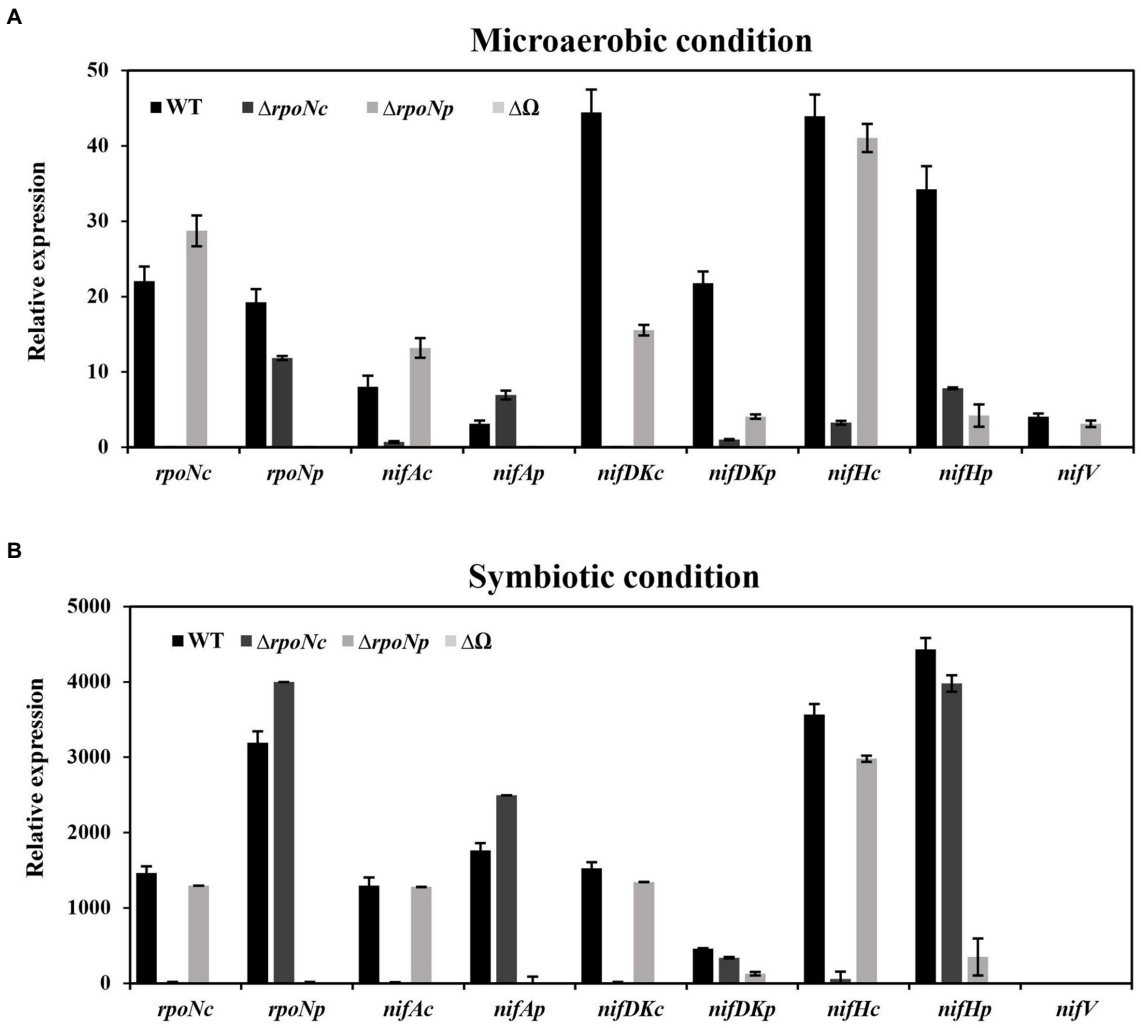
Expression of nitrogen fixing genes (*rpoNc*, *rpoNp*, *nifAc*, *nifAp*, *nifDKc*, *nifDKp*, *nifHc*, *nifHp*, and *nifV*) in DOA9 WT and Δ*rpoNc*, Δ*rpoNp*, and Δ*rpoNp*::Ω*rpoNc* (ΔΩ) mutant strains under free-living **(A)** and symbiotic conditions **(B)**. Total RNA was extracted and expression of *nif* genes was determined by quantitative reverse transcription-PCR (qRT-PCR) using 16S RNA as internal standard. Results are the average of three independent replicates.

The results presented thus far indicate that deletion of *rpoNc* and/or *rpoNp* has an impact on the nitrogenase enzyme activity under free-living and symbiotic conditions. To investigate the direct impact of deletion *rpoNc* and *rpoNp* on the expression of *nif* genes, we have determined the expression of *nifAc*, *nifAp*, *nifDK*c, *nifDK*p, *nifHc*, *nifHp*, and *nifV* in the different *rpoN* mutants by qRT-PCR. The results showed that under free-living nitrogen fixing conditions, the deletion of *rpoNc* had a very drastic negative effect on the expression of the majority of the tested chromosomal encoded *nif* genes (Figure 6A). In contrast, deletion of the *rpoNp* gene resulted only in a small reduction of plasmid encoded *nif* gene expressions, whereas a complete absence of *nif* gene expression was observed with the double DOA9Δ*rpoNp*::Ω*rpoNc* mutant. To analyze *nif* gene expression under symbiotic conditions, nodules of plants inoculated with the different *rpoN* mutants were collected at 21 dpi. qRT-PCR analysis showed that for all *rpoN* mutants, the *nif* gene expression profile in nodules resembled those as observed in free-living conditions (Figure 6B).

## 4. Discussion

*Bradyrhizobium* sp. strain DOA9 is an example of a diazotrophic bacterium that has the ability to fix nitrogen under both free-living and symbiotic conditions. DOA9 contains two sets of structural *nifHDK* and *nifA* regulator genes: one localized on the chromosome and a second localized on a mega-plasmid. Previous studies showed that during symbiosis, the two *nifDK* copies contribute differently to nitrogenase enzyme activity, whereas the two *nifA* copies are functionally redundant (Wongdee et al., 2016, 2018). In diazotroph bacteria, the NifA protein associates with the RNA polymerase sigma factor RpoN (sigma 54) to activate *nif* gene expression. As *Bradyrhizobium* sp. DOA9 also contains a chromosomal and plasmid-encoded RpoN protein, we have inactivated these two *rpoN* genes and analyzed the effect on free-living and symbiotic conditions.

In diazotrophic bacteria the most well-known RpoN activity (together with NifA) is the regulation of the expression of *nif* genes. Expression studies using GusA reporter strains showed that *rpoNc* and *rpoNp* were almost equally expressed under both free-living and symbiotic conditions (Figures 2A,B). However, only inactivation of *rpoNc* had a drastic effect on the nitrogenase activity of cells grown under free-living conditions (Figure 4). The absence of nitrogenase enzyme activity with the *rpoNc* mutant correlates with the almost complete absence of chromosomal encoded *nif* gene expression in this mutant under free-living conditions (Figure 6A). Previously, we have shown that chromosomal encoded NifDKc and NifAc are important for free-living nitrogen enzyme activity (Wongdee et al., 2016, 2018). Together with the results presented here, this indicates that under free-living conditions, NifAc and RpoNc are responsible for the expression of the chromosomal encoded *nif* genes needed for nitrogen fixation.

The importance of RpoNc and RpoNp in nitrogen fixation during symbiosis seems more complex. Inactivation of single copies of chromosomal and plasmid-encoded NifDK and NifA have only a slight effect or none at all on the symbiosis and symbiotic nitrogenase enzyme activity (Wongdee et al., 2016, 2018). However, inactivation of individual copies of *rpoNc* and *rpoNp* have a very drastic effect on nodulation and symbiotic nitrogen fixation. Induction of *nif* gene expression is one of the last stages in a functional symbiotic interaction. Analysis of the *rpoN* mutants under free-living conditions showed that individual inactivation of chromosomal and plasmid-encoded *rpoN* has an impact on colony morphology, swimming mobility, utilization of C and N sources, EPS production, and biofilm formation. Although the effect of *rpoNc* inactivation often had a bigger impact than *rpoNp* inactivation, most observed differences seemed to impact the bacterial cell surface.

Analysis of the nodule numbers induced by *rpoN* mutants on *A. americana* plants showed that they are drastically reduced in comparison to plants inoculated with WT DOA9. In general, rhizobia mutants that are unable to form an active nitrogenase enzyme complex induce more nodules on leguminous plants than the WT, as previously observed with DOA9 *nifDK* mutants in *A. americana* (Wongdee et al., 2016). Therefore, the reduced nodule number obtained with all *rpoN* mutants indicates that the symbiotic interaction is already disturbed at an early stage. Therefore, we hypothesize that the alterations observed in all *rpoN* mutants under free-living conditions also impact the early stages of the symbiotic interaction with *A. americana* plants and that they are directly responsible for the reduced nodule number and nitrogenase activity observed.

Interestingly, alterations such as colony morphology and EPS observed for DOA9 *rpoN* mutants under free-living conditions were not observed when we tested the different DOA9 *nifA* mutants (Supplementary Figure S6). This suggests that RpoNc and RpoNp regulate genes involved in colony morphology, swimming mobility, utilization of C and N sources, EPS production, and biofilm formation independently of NifAc and NifAp. The RpoN-dependent regulation of other processes than *nif* genes seems not to be unique for *Bradyrhizobium* sp. DOA9. Such RpoN-dependent regulation has also been reported for other symbiotic bacteria (*B. japonicum*, *S. meliloti*, *R. etli*, and *Mesorhizobium loti*) (Kullik et al., 1991; Michiels et al., 1998; Sullivan et al., 2013) and some pathogenic bacteria (*E. coli*, *Listeria monocytogenes*, *Salmonella enterica*, *Pseudomonas aeruginosa*, *Burkholderia cenocepacia*, *Vibrio anguillarum* and *Ralstonia solanacearum*) (Arous et al., 2004; Dong and Mekalanos, 2012; Cai et al., 2015; Lundgren et al., 2015; Fazli et al., 2017; Riordan and Mitra, 2017). RpoN needs the binding of an activator (regulator) protein to initiate transcription. As NifAc and NifAp are only regulating *nif* gene expression in DOA9 other “activator-RpoN” couples are expected to be involved in the regulation of genes involved in EPS production and biofilm formation. Previous research has shown that the activator proteins interacting with RpoN contain an RNA polymerase sigma factor 54 interaction domain (IPR002078). When we searched the chromosome and plasmid of the DOA9 strain for proteins containing an IPR002078 domain, we found 11 regulator proteins (NifAc, NifAp, HoxAc, HoxAp, BRADOA9_v1_20290c, BRADOA9_v1_21693c, BRADOA9_v1_40641c, FlbDc, GlnGc (NtrCc), NtrXc, BRADOA9_v1_50924c) that putatively could interact with RpoN. Interestingly, three of these regulator proteins, GlnG (NtrC), NtrX and FlbD have been shown to be involved in EPS production, and biofilm formation in other bacteria (Kim et al., 2009; Calatrava-Morales et al., 2017; Dardis et al., 2021). Future studies are necessary to analyse if the *in silico* identified proteins indeed interact with RpoNc and/or RpoNp and if these are implicated in the different phenotypes as observed in this study.

## Data availability statement

The original contributions presented in the study are included in the article/Supplementary material, further inquiries can be directed to the corresponding authors.

## Ethics statement

The biosecurity concerns of this study were reviewed and approved by Suranaree University of Technology (approval number: SUT-IBC-05/2021) and are in accordance with the levels of risk in pathogens andanimal toxins listed in “the Risk Group of Pathogen and Animal Toxin (2017)” issued by the Department of Medical Sciences, Ministry of Public Health, the Pathogen and Animal Toxin Act (2015) and Biosafety Guidelines for Modern Biotechnology, BIOTEC (2016).

## Author contributions

JW, NN, NT, PT, and EG conceived and conducted the experiments. JW, NN, PT, and EG analyzed the results and wrote the manuscript. All authors reviewed the manuscript.

## Funding

This work was supported by (I) Suranaree University of Technology (SUT), (II) Thailand Science Research and Innovation (TSRI), (III) National Science, Research, and Innovation Fund (NSRF) (code 160347), (IV) Program Management Unit for Human Resources and Insititutional Development and Innovation (PMU-B) grant number B16F640113, (V) IRD (JEAI_Symbi Trop), and (VI) The Franco-Thai Cooperation program in Higher Education and Research (PHC SIAM 2022-No.48151XL).

## Conflict of interest

The authors declare that the research was conducted in the absence of any commercial or financial relationships that could be construed as a potential conflict of interest.

## Publisher’s note

All claims expressed in this article are solely those of the authors and do not necessarily represent those of their affiliated organizations, or those of the publisher, the editors and the reviewers. Any product that may be evaluated in this article, or claim that may be made by its manufacturer, is not guaranteed or endorsed by the publisher.

## Footnotes

^1^ https://mage.genoscope.cns.fr/microscope/home/index.php
