# Supplementary material for: Role of two RpoN in Bradyrhizobium sp. strain DOA9 in symbiosis and free-living growth
Source: Front Microbiol. 2023 Feb 16;14:1131860. doi: 10.3389/fmicb.2023.1131860 (PMC9977809; doi:10.3389/fmicb.2023.1131860)
Supplement: Supplementary file 1 [file Data_Sheet_1.PDF]

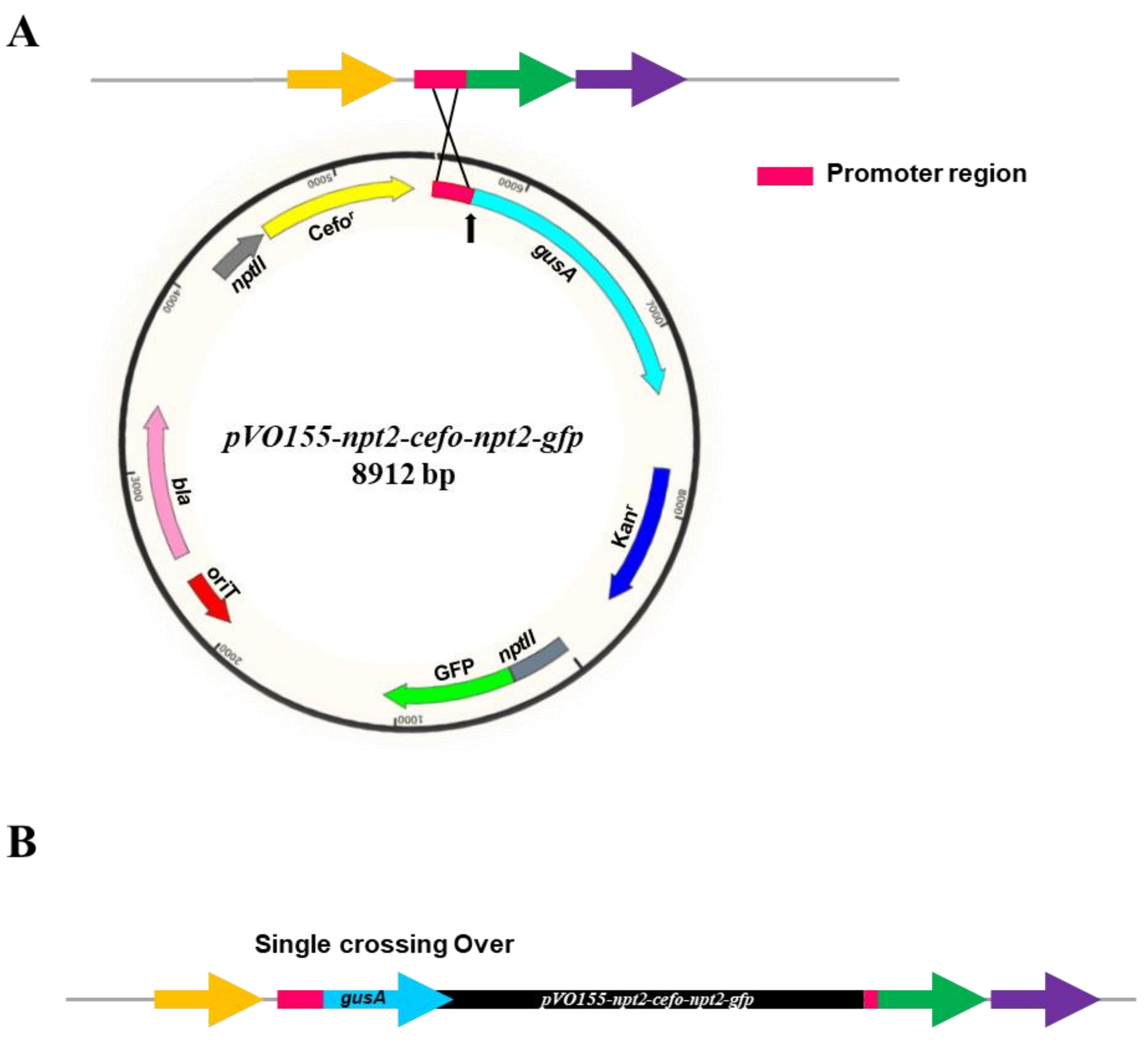

Figure S1. Schematic overview of promoter GusA-fusion of selected gene. A +/-400-base-pair (bp) upstream region (Pink fragment) of selected gene (Green fragment) was amplified by PCR, and the corresponding DNA fragment was cloned into plasmid pVO155-*npt2-cefo-npt2-gfp* (Black arrow). This plasmid cannot replicate in *Bradyrhizobium* contains promoterless *gusA* gene, and kanamycin (Kan^r) resistance gene, *gfp* gene under control of the constitutive *nptII* promoter (GFP), origin for transfer (oriT), beta-lactamases (*bla*) and cefotaxime resistance gene ($Cefo^r$). The plasmid was introduced into *Bradyrhizobium* sp. DOA9 via conjugation and clones in which the plasmid was inserted into the target region via single crossing over (**A**). Isolated clones were verified by sequencing, and good clones were selected as an expected gene arrangement into target DNA region (**B**).

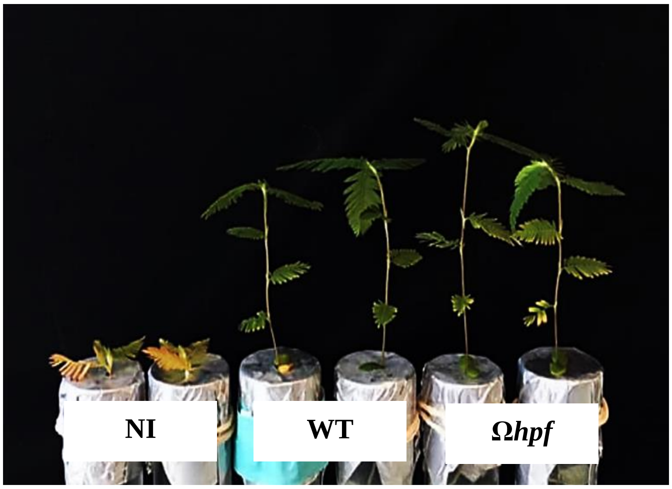

Figure S2. *Aeschynomene americana* plants grown under nitrogen-limiting conditions and inoculated with *Bradyrhizobium* sp. DOA9 wild-type (WT) and the *hpf* insertion mutant (Ω*hpf*) at 21 dpi. As inactivation of the *hpf* gene has no effect on the symbiotic interaction of *Bradyrhizobium* DOA9 with *A. americana*, the result demonstrate that the symbiotic defect observed with the DOA9 *rpoNc* mutant is not due to a polar effect on the downstream localized *hpf* gene. The result indicated no effect of *hpf* gene on symbiosis, and there was no polar effect of *rpoN* mutagenesis.

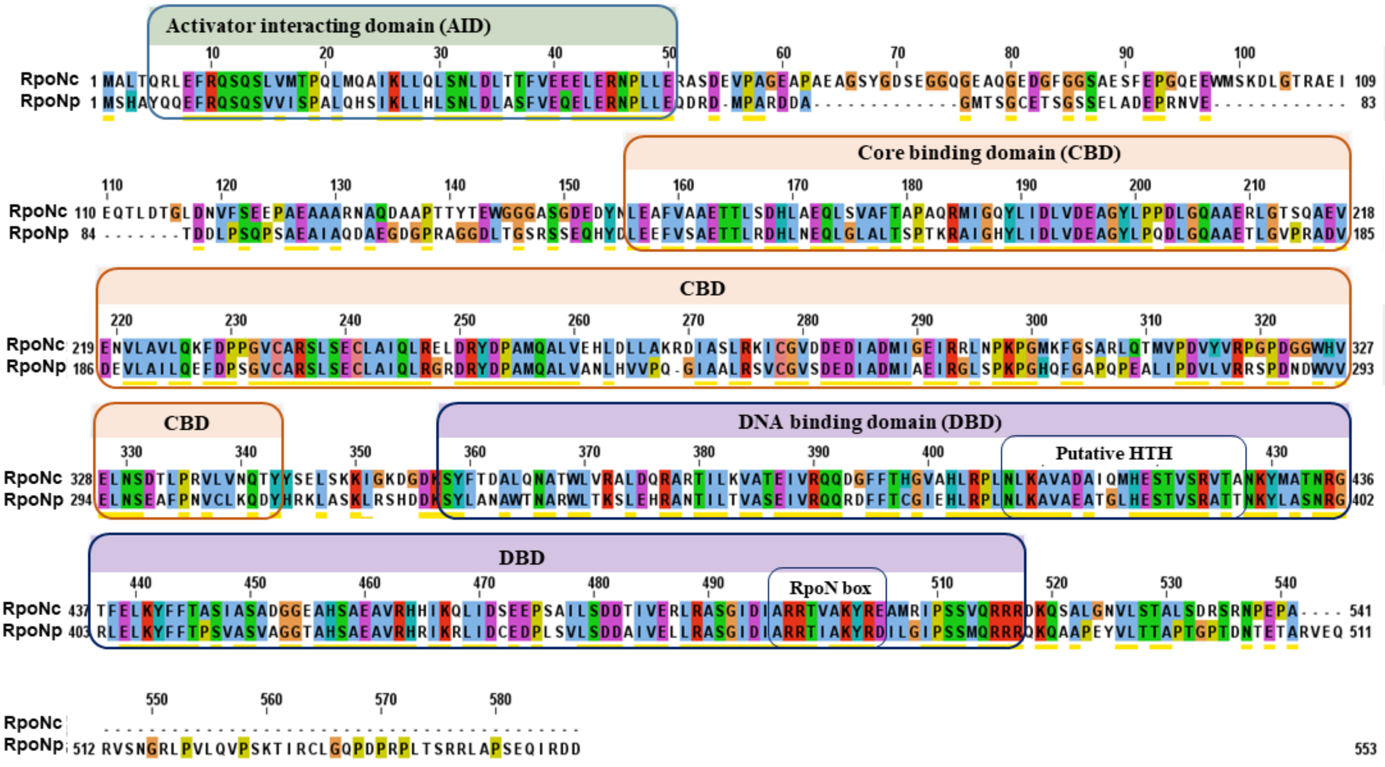

Figure S3. Clustal W amino acid (aa) sequence alignment of *Bradyrhizobium* sp. DOA9 RpoNc (WP_025032974.1) and RpoNp (WP_082847983.1). Note the large deletions (positions 62-77 and 98-118) present in the region between the AID and CBD domain and C-terminal extension (position 542-587) in the RpoNp protein.

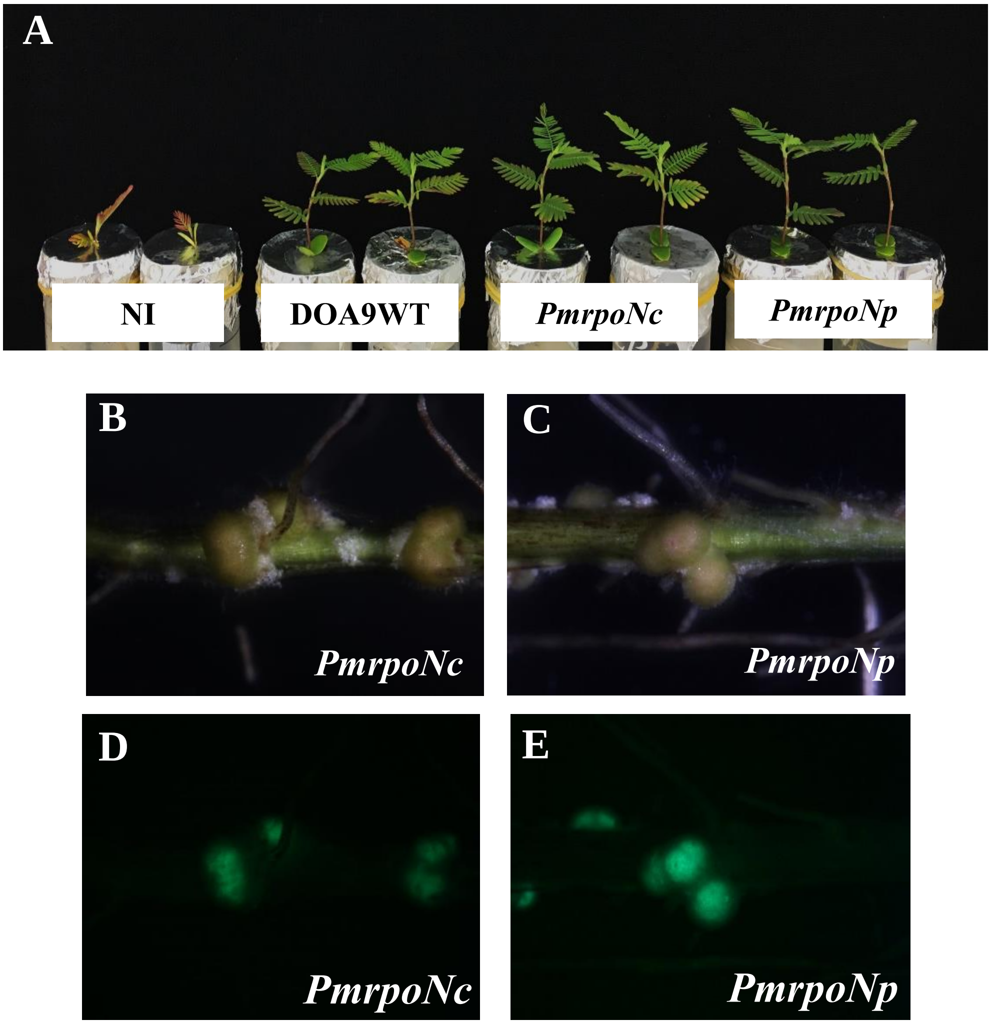

Figure S4. *Aeschynomene americana* plants grown under nitrogen limiting conditions and inoculated with wild-type *Bradyrhizobium* sp. DOA9 (WT) and DOA9 *PmrpoNc* and *PmrpoNp* GusA reporter strains at 21 dpi (**A**). Nodules on roots of *A. americana* plants inoculated with the *PmrpoNc* and *PmrpoNp* GusA reporter strains at 21 dpi (**B and C**). GFP fluorescence of nodules on plants inoculated with *PmrpoNc* and *PmrpoNp* reporter strains (**D and E**).

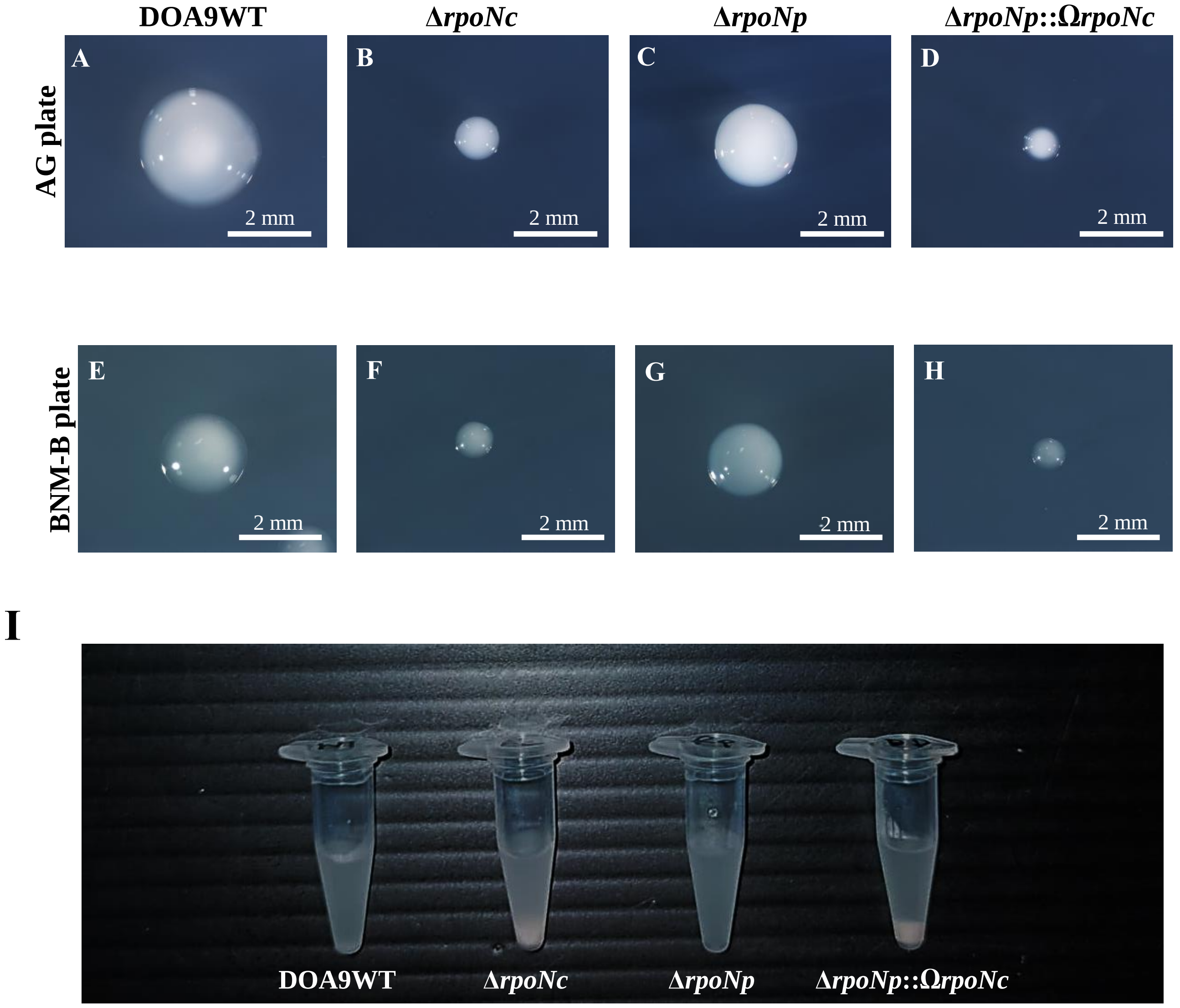

Figure S5. Colony morphology of DOA9WT and DOA9Δ*rpoNc*, DOA9Δ*rpoNp* and DOA9Δ*rpoNp*::Ω*rpoNc* mutants on AG agar plates at 7 days (**A-D**) and BNM-B agar plates at 9 days (**E-H**). Pellet of bacterial cells scraped from agar plates, dissolved in deionized water and centrifuged for 15 seconds in an Eppendorf centrifuge tube (**I**).

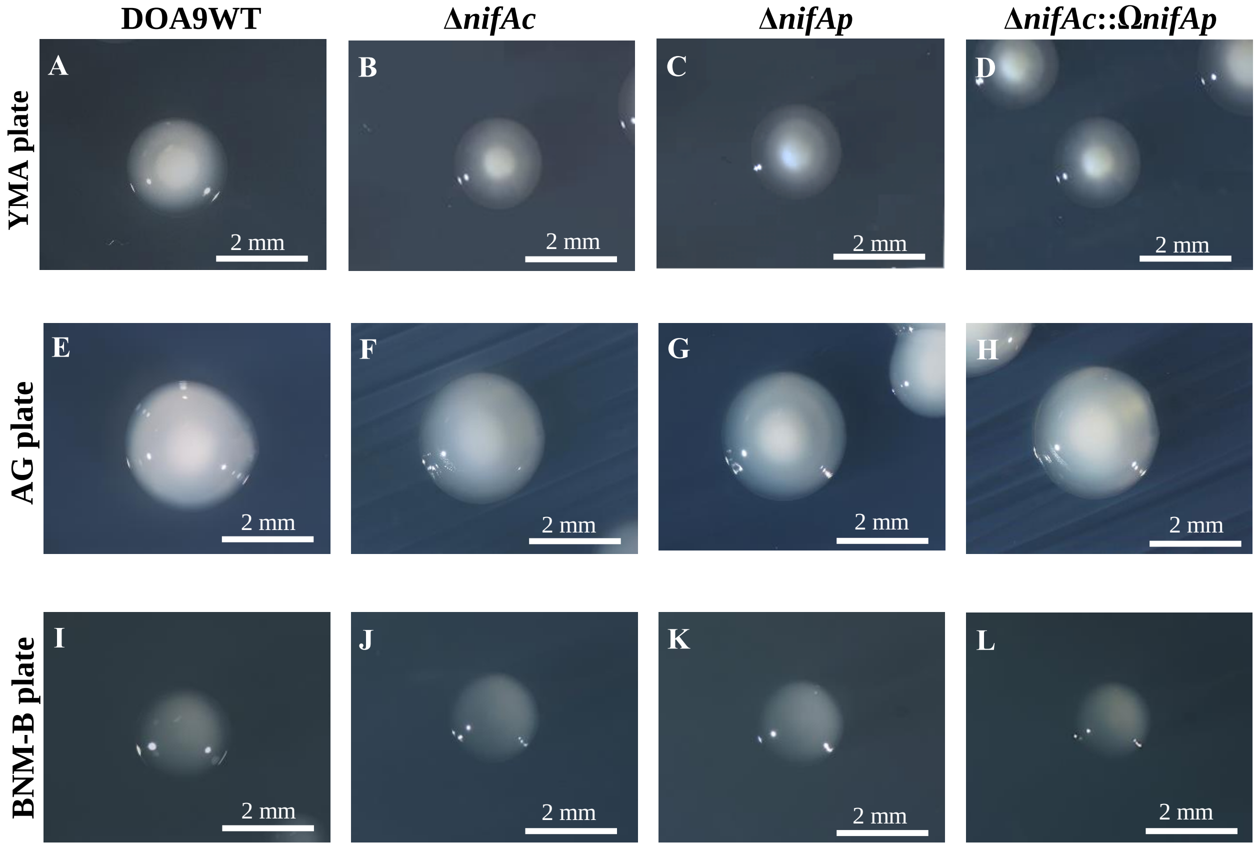

Figure S6. Colony morphology of DOA9WT and mutants; DOA9Δ*nifAc*, DOA9Δ*nifAp*, and DOA9Δ*nifAc*::Ω*nifAp* were grown on YEM (**A-D**) and AG (**E-H**) plates at 7 days and on BNM-B plate at 9 days (**I-L**).

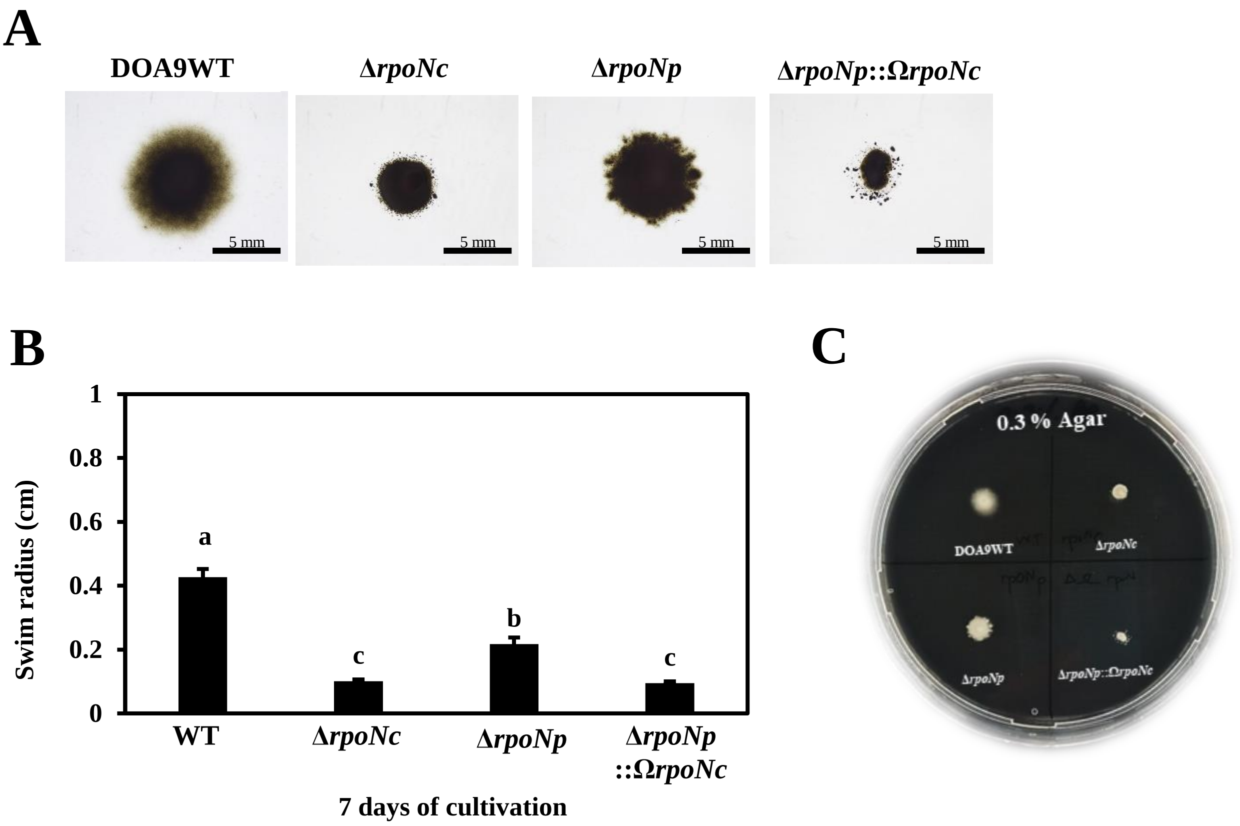

Figure S7. Swimming motility test of DOA9WT, DOA9Δ*rpoNc*, DOA9Δ*rpoNp*, and DOA9Δ*rpoNp*::Ω*rpoNc* strains. Bacterial cell spots with translucent background of WT and all *rpoN* mutant strains in semi-solid AG medium containing 0.3% (w/v) agar after 7 days of cultivation at 28ºC (**A**). The spot diameter in the swimming motility test was measured and calculated as the swim radius (cm) from 5 replicates (**B**). Comparison of bacterial growth in semi-solid AG medium that was stabbed with 10^8 CFU/ml using sterilized toothpick (**C**).

A

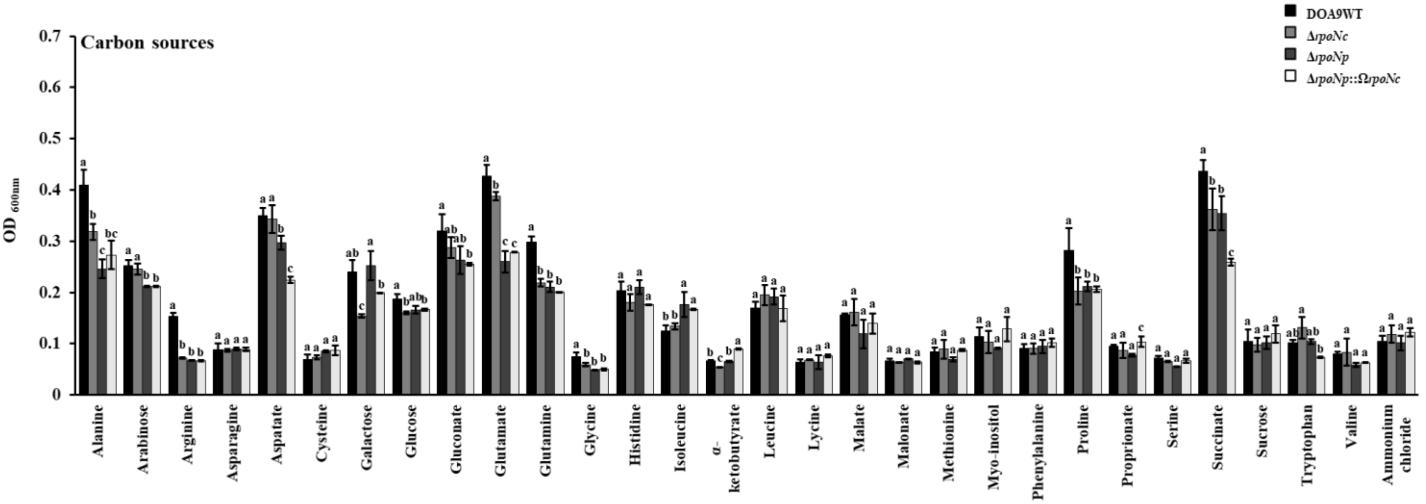

B

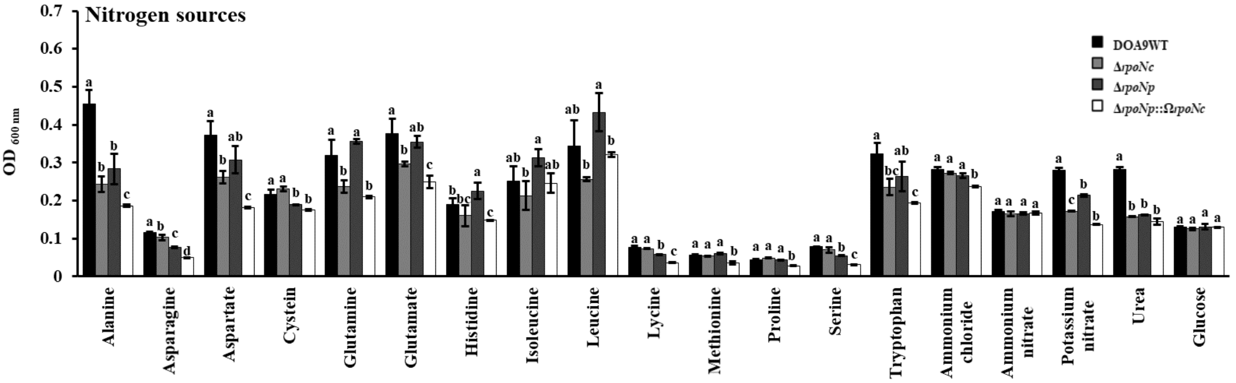

Figure S8. Aerobic growth of *Bradyrhizobium* sp. strain DOA9WT and *rpoN* mutants in BNM-B medium containing different carbon (**A**) and nitrogen sources (**B**). The bacterial growth was determined (OD600) after 10 days at 28°C Ammonium chloride (NH_4Cl; 10 mM) and glucose (20 mM) were added to the growth medium to test the utilization of different carbon and nitrogen sources, respectively. Data sets represent mean values of 3 replications and statistically significant differences (ANOVA Tukey test (p<0.01)) are indicated.

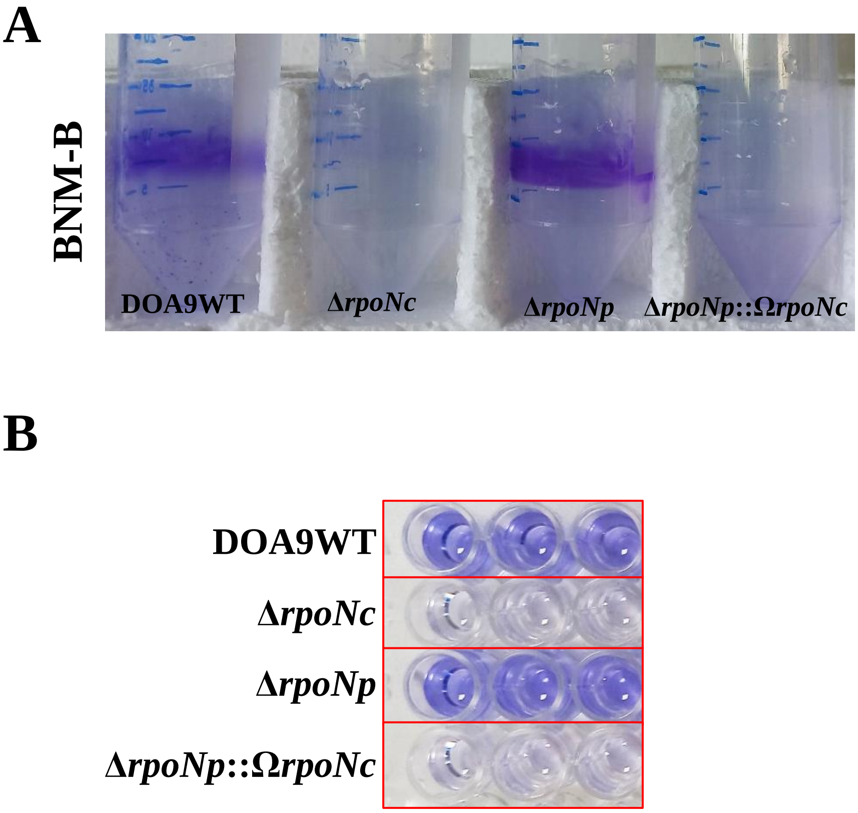

Figure S9. Biofilm formation of DOA9WT and *rpoN* mutants in BNM-B medium supplemented with 20 mM succinate. Biofilm formation in WT and *rpoN* mutant cultures was determined by staining the Falcon tubes with 0.01 % of crystal violet for 15 minutes (**A**). Solubilisation the crystal violet stain (3 replicates) with ethanol before measuring the optical density at 570 nm (**B**).

Table S1. Primers used in this study.

Primers	Sequences (5' → 3')	Relevant characteristics
Pm.rpoN-DOA9c.f<br>Pm.rpoN-DOA9c.r	CATCC**GTCGAC**TCGTCCGCCATCTGACCAATC/<br>AATCT**TCTAGA**AAGCGCCATGAAGCAAGAACC	Cloning (SalI/XbaI) promoter region *PmrpoNc* into pVO155-Cefo-npt2-gfp. Plasmid is used to construct the GusA-reporter strain DOA9Pm*rpoNc*
PmRpoN-DOA9p.f<br>PmRpoN-DOA9p.r	CTGCG**GTCGAC**CGTCGCAAAACTACCTGCAC/<br>CCTGT**TCTAGA**GCATGGCTCATTGAAACACCTTC	Cloning (SalI/XbaI) promoter region *PmrpoNp* into pVO155-Cefo-npt2-gfp. Plasmid is used to construct the GusA-reporter strain DOA9*PmrpoNp*
up.rpoN.DOA9c.f/<br>up.rpoN.DOA9c.r	GCGCCG**GGATCC**CCACCGTGTTCTACATGATCAC/<br>TCAGGCCGGTTC**AAGCTT**CGTAAGCGCCATGAAG CAAGAACC<br>GAACCGGCCTGA	Amplification of the upstream region of *rpoNc* to construct a large *rpoNc* deletion by overlapping PCR. The region homologous to the downstream region is underlined.
dw.rpoN.DOA9c.f/<br>dw.rpoN.DOA9c.r	ATGGCGCTTACG**AAGCTT**GAACCGGCCTGATTGC GCTGGCGCCAA/<br>TTAGTCGTCTCT**AAGCTT**ATATGCATGGCTCATTG AAACACC	Amplification of the downstream region of *rpoNc* to construct a large *rpoNc* deletion by overlapping PCR. The region homologous to the upstream region is underlined. After fusion with the upstream region, the PCR product was cloned into plasmid pK18mob-cefo-sacB (BamHI/XbaI). Plasmid is used to construct the DOA9Δ*rpoNc* mutant strain
up.rpoN.DOA9p.f/<br>up.rpoN.DOA9p.r	TGTTGT**GGATCC**CGCCTTGCTGAGTCCTTAACTG/<br>TTAGTCGTCTCT**AAGCTT**ATATGCATGGCTCATTG AAACACC	Amplification of the upstream region of *rpoNp* to construct a large *rpoNp* deletion by overlapping PCR. The region homologous to the downstream region is underlined.
dw.rpoN.DOA9p.f/<br>dw.rpoN.DOA9p.r	AGCCATGCATAT**AAGCTT**AGAGACGACTAACCGG CCCTAAGTCAC/<br>ACGGGC**TCTAGA**TCCATCAAAGCACGATAATAAG	Amplification of the downstream region of *rpoNp* to construct a large *rpoNp* deletion by overlapping PCR. The region homologous to the upstream region is underlined. After fusion with the upstream region, the PCR product was cloned into plasmid pK18mob-cefo-sacB (BamHI/XbaI). Plasmid is used to construct the DOA9Δ*rpoNp* mutant strain
RpoN.DOA9c.in.f/<br>RpoN.DOA9c.in.r	CGCAG**GTCGAC**CAGGCGATCAAGCTGCTGCAATT G<br>CGTCAC**TCTAGA**CGCCGCCGCCCCATTCGGTATAG	Cloning (SalI/XbaI) of a 328 bp internal *rpoNc* fragment in pVO155-Cefo-npt2-gfp. The plasmid is used to construct the Δ*rpoNp*::Ω*rpoNc* mutant strain

Table S2. Analysis of NifA-RpoN binding-site sequences located upstream of the nitrogen-fixing genes on both chromosome (c) and mega-plasmid (p) of *Bradyrhizobium* sp. strain DOA9.

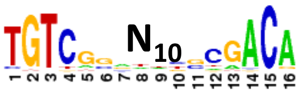

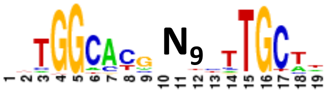

nif genes	NifA-binding box	RpoN-binding box
nifAc	TGTCGCGAACTCGACA	TGGCCCAATTCCTGC
nifAp	TGTCGATCGCAGAACA	TGGGATGCAACATTC
rpoNc	TGTCAGGGATGCGCCG	CGGCTTCCTGGCTGT
rpoNp	TGTCCGACACCATACA	TGGCACGCGCCTTGC
nifDKc	TGTTTTGTTTCGAACA	TGGCACACTCGTTGC
nifDKp	TATCGGGTTTGCGACA	CGGCACAGTCCTTGC
nifHc	TGTCCGGTTTCTGACA	TGGCACGCCAGTTGC
nifHp	TGTCTGATACCTAATA	CGGCACATCAATTGC
